# Marine Reserves and Reproductive Biomass: A Case Study of a Heavily Targeted Reef Fish

**DOI:** 10.1371/journal.pone.0039599

**Published:** 2012-06-26

**Authors:** Brett M. Taylor, Jennifer L. McIlwain, Alexander M. Kerr

**Affiliations:** 1 Marine Laboratory, University of Guam, Mangilao, Guam, United States of America; 2 School of Marine and Tropical Biology, James Cook University, Townsville, Queensland, Australia; 3 Department of Environment and Agriculture, Curtin University, Perth, Western Australia, Australia; 4 Australian Research Council Centre of Excellence for Coral Reef Studies, Townsville, Queensland, Australia; Aristotle University of Thessaloniki, Greece

## Abstract

Recruitment overfishing (the reduction of a spawning stock past a point at which the stock can no longer replenish itself) is a common problem which can lead to a rapid and irreversible fishery collapse. Averting this disaster requires maintaining a sufficient spawning population to buffer stochastic fluctuations in recruitment of heavily harvested stocks. Optimal strategies for managing spawner biomass are well developed for temperate systems, yet remain uncertain for tropical fisheries, where the danger of collapse from recruitment overfishing looms largest. In this study, we explored empirically and through modeling, the role of marine reserves in maximizing spawner biomass of a heavily exploited reef fish, *Lethrinus harak* around Guam, Micronesia. On average, spawner biomass was 16 times higher inside the reserves compared with adjacent fished sites. Adult density and habitat-specific mean fish size were also significantly greater. We used these data in an age-structured population model to explore the effect of several management scenarios on *L. harak* demography. Under minimum-size limits, unlimited extraction and all rotational-closure scenarios, the model predicts that preferential mortality of larger and older fish prompt dramatic declines in spawner biomass and the proportion of male fish, as well as considerable declines in total abundance. For rotational closures this occurred because of the mismatch between the scales of recovery and extraction. Our results highlight how alternative management scenarios fall short in comparison to marine reserves in preserving reproductively viable fish populations on coral reefs.

## Introduction

Despite the burgeoning scientific literature demonstrating the effectiveness of marine reserves as fishery management tools, protected-area management continues to be controversial in some areas. This is due to legitimate concerns by fishing interest groups that protected areas displace fishing effort and reduce overall yield [1]. Such concerns may be valid but depend on the type and status of the fishery in question [2]. Alternative management such as minimum size limits has been effective in highly regulated fisheries and rotational area closures have been valuable for sedentary invertebrate stocks [3]. For coral reefs and associated habitats, however, permanent marine reserves are now considered practical and effective fishery policy, especially for heavily-exploited, multi-species fisheries [4], [5], [6], [7]. The long-term, economic benefits of coral reef reserves are only beginning to be realized with significantly greater income for fishers adjacent to closed areas [8].

Reserves benefit fisheries through the net export of fish via larval production or adult movement, thereby offsetting the increased fishing effort from displaced fishermen and buffering against recruitment overfishing [1], [9], [10], [11]. Increased larval production from marine reserves occurs as larger, older fish, who often contribute disproportionately to egg production [12], [13], accumulate in the population [14]. This build-up of older individuals has added benefits as larval quality and survivorship increases considerably with maternal age [15]. Direct measures of reproductive potential (e.g. spawner biomass) within reserves are seldom made, but recent predictions suggest larval export from reserves is greatest when the differential in production between protected and fished sites is very large [11].

When making predictions about reproductive potential and marine reserves at the population level, it is essential to build robust models that combine life-history and demographic data. An early but comprehensive model of the effects of protected areas on cod populations demonstrated total spawning stock biomass of a population increases drastically with the implementation of reserves when transfer rates between protected and fished areas are low [14]. An extension of this model to multiple tropical reef-fish species across an array of life histories reached the same conclusion [16]. However, the latter study was based on erroneous estimates of growth, longevity, and mortality for some of the species types modeled [17], [18], [19], resulting in underestimates of longevity and overestimates of natural mortality. This is expected to have a major influence on the dynamics of build-up inside protected areas and the subsequent estimates of yield and spawner biomass [20]. Today, we still lack an estimate of the contribution that various management scenarios have in preserving spawning stock biomass in coral-reef fisheries.

The potential for marine reserves to safeguard against recruitment overfishing [the depletion of an adult spawning stock past the point where it no longer has the capacity to replenish itself] is largely dependent on life-history characteristics of the protected species [20]. Important parameters include growth rate, longevity, natural mortality, size at maturity, and vagility. The thumbprint emperor, *Lethrinus harak*, is a common food fish throughout Indo-Pacific reefs. It is also an ideal study subject for evaluating small-scale fishing effects because it is often abundant, common across multiple habitats, and amenable to quantify with visual surveys. Further, several studies from the west Pacific have described life-history features of the species, demonstrating a moderate lifespan of 13 to 14 years [21], [22], relatively slow growth and late maturation [21], protogynous hermaphroditism [23], monthly spawning at aggregation sites [24], and small home ranges [25]. Many of these traits are known to increase a species’ vulnerability to overexploitation and obtaining such data is a necessary first step in evaluating management strategies.

Here, we test the assumption that marine reserves contribute disproportionately to regional reproductive biomass of the heavily exploited reef fish, *Lethrinus harak*. We employ an optimal stratified-random survey design at four sites; two protected and two unprotected from fishing. At each site we estimate total abundance, habitat-specific density, size frequency, and biomass. These demographic data were combined with life-history data [21] to construct an age-structured population model from which predictions about population size and structure were made using alternative management scenarios.

**Figure 1:**
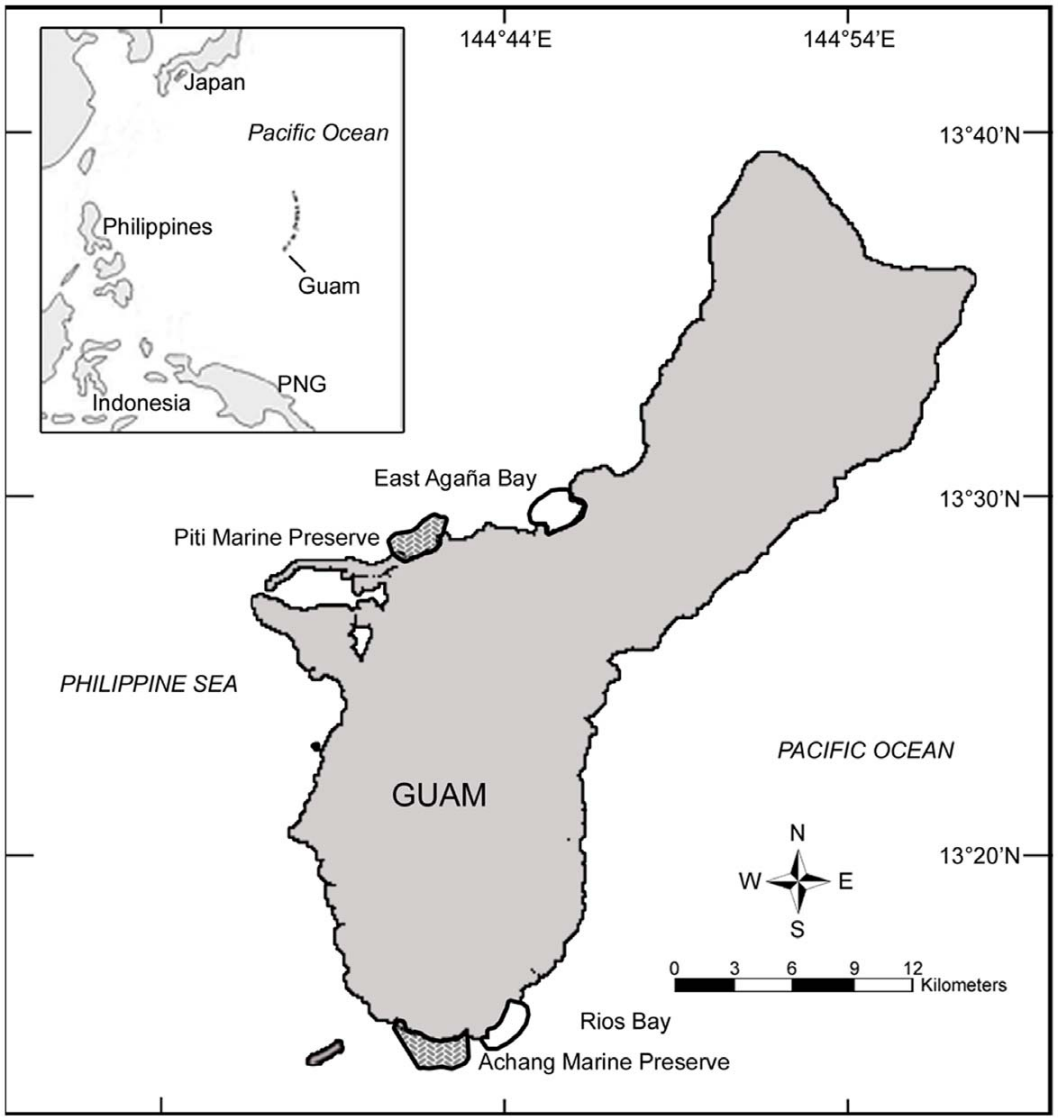
Location of study sites on Guam. Protected sites include Piti Marine Preserve and Achang Marine Preserve. Fished sites include East Agaña Bay and Rios Bay.

**Figure 2:**
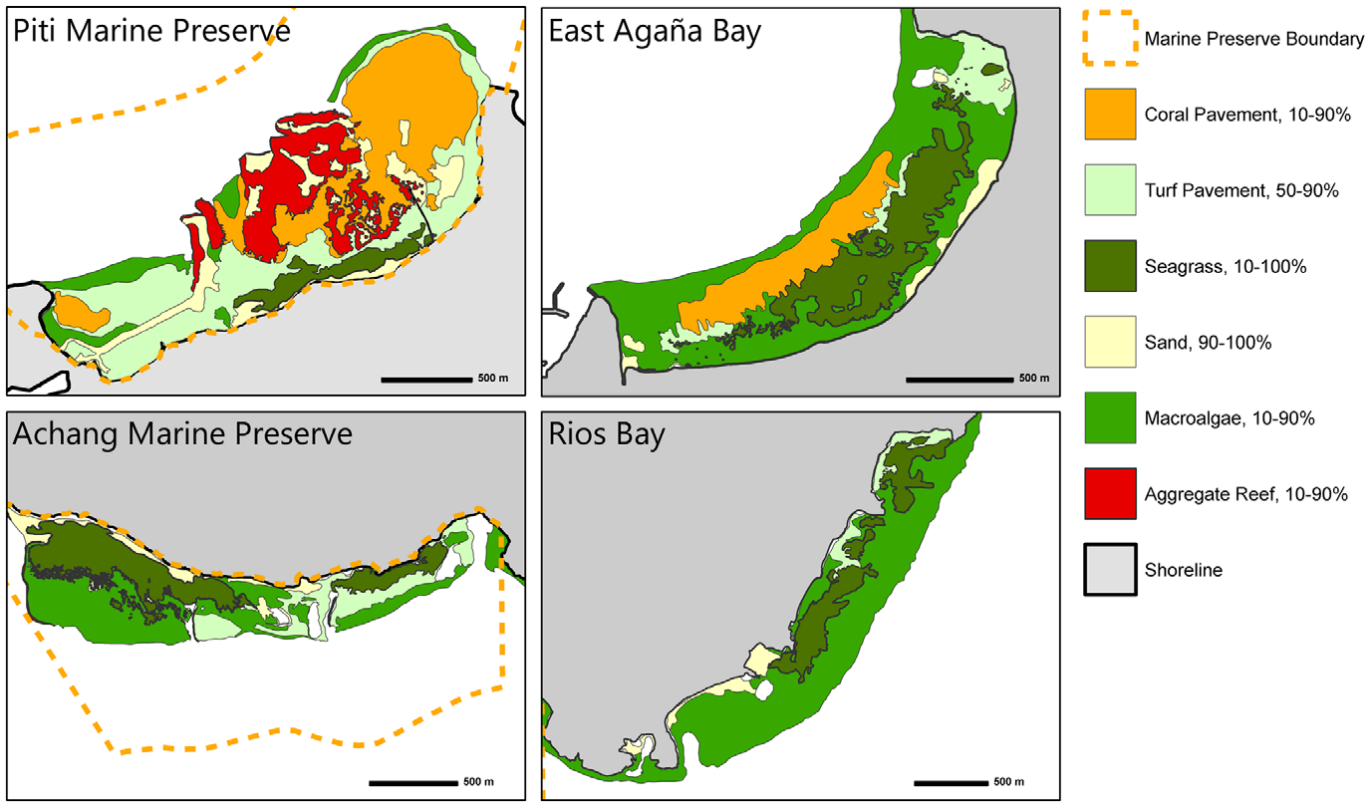
Benthic habitat maps of the reef flat at Piti Marine Preserve, East Agaña Bay, Achang Marine Preserve, and Rios Bay. The distribution of the six primary habitat types are color-coded.

**Table 1:** Total abundance of *L. harak* at each site estimated from an optimal stratified-random sampling design.

Piti Marine Preserve										
Strata	Area (m^2^)	% Area	N_h_	W_h_	n_h_	x_h_	s_h_^2^	(W^2^_h_*s^2^_h_)/n_h_	N_h_*x_h_	% Fish
Aggregate Reef	308 341	20.2	1233.4	0.202	12	2.25	2.568	0.00875	2775.1	25.9
Coral pavement	474 762	31.1	1899.0	0.311	36	2.86	8.523	0.02296	5433.4	50.7
Macroalgae	37 008	2.4	148.0	0.024	8	3.63	9.411	0.00069	536.6	5.0
Seagrass	85 276	5.6	341.1	0.056	20	3.90	12.200	0.00191	1330.3	12.4
Turf pavement	431 191	28.3	1724.8	0.283	6	0.00	0.000	0.00000	0.0	0.0
Sand	187 981	12.3	751.9	0.123	7	0.86	0.476	0.00103	644.5	6.0
Totals	1 524 559		6098.2	1.000	n = 89		s^2^(x_strat_) =	0.03535	10 719.9	
**East Agaña Bay**										
**Strata**	**Area (m^2^)**	**% Area**	**N_h_**	**W_h_**	**n_h_**	**x_h_**	**s_h_^2^**	(W^2^_h_*s^2^_h_)/n_h_	N_h_*x_h_	**% Fish**
Coral pavement	287 399	16.7	1149.6	0.167	5	1.20	1.700	0.00947	1379.5	7.5
Macroalgae	830 580	48.2	3322.3	0.482	10	0.44	0.266	0.00619	1461.8	8.0
Seagrass	371 768	21.6	1487.1	0.216	65	10.05	425.701	0.30533	14 939.4	81.5
Turf pavement	170 694	9.9	682.8	0.099	5	0.80	1.700	0.00334	546.2	3.0
Sand	61 351	3.6	245.4	0.036	5	0.00	0.000	0.00000	0.0	0.0
Totals	1 721 793		6887.2	1	n = 90		s^2^(x_strat_) =	0.32434	18 326.9	
**Achang Marine Preserve**										
**Strata**	**Area (m^2^)**	**% Area**	**N_h_**	**W_h_**	**n_h_**	**x_h_**	**s_h_^2^**	(W^2^_h_*s^2^_h_)/n_h_	N_h_*x_h_	**% Fish**
Macroalgae	251 940	17.9	1007.8	0.179	11	1.00	3.400	0.00993	1007.8	2.1
Seagrass	486 249	34.6	1945.0	0.346	31	20.55	158.056	0.61009	39 966.5	82.3
Turf pavement	597 353	42.5	2389.4	0.425	36	2.40	18.894	0.09478	5734.6	11.8
Sand	70 140	5.0	280.6	0.050	5	6.60	8.800	0.00438	1851.7	3.8
Totals	1 405 681		5622.7	1	n = 83		s^2^(x_strat_) =	0.71918	48 560.5	
**Rios Bay**										
**Strata**	**Area (m^2^)**	**% Area**	**N_h_**	**W_h_**	**n_h_**	**x_h_**	**s_h_^2^**	(W^2^_h_*s^2^_h_)/n_h_	N_h_*x_h_	**% Fish**
Macroalgae	647 667	69.7	2590.7	0.697	17	1.94	15.559	0.44436	5029.0	73.1
Seagrass	184 470	19.8	737.9	0.198	27	2.44	12.026	0.01754	1803.7	26.2
Turf pavement	53 805	5.8	215.2	0.058	5	0.20	0.200	0.00013	43.0	0.6
Sand	43 553	4.7	174.2	0.047	5	0.00	0.000	0.00000	0.0	0.0
Totals	929 496		3718.0	1	n = 54		s^2^(x_strat_) =	0.46204	6875.7	

N_h_ represents the theoretical maximum number of transects that could be fit into habitat *h* without overlap, W_h_ is the proportional habitat area, n_h_ is the number of transects allocated to habitat *h*, x_h_ is the mean number of fish per transect for habitat *h*, s_h_^2^ is the variance surrounding the density estimate for habitat *h*, *n* is the total number of transects at a site, and s^2^(x_strat_) is the variance surrounding the stratified mean density for the entire site.

## Materials and Methods

### Ethics Statement

All research was conducted under the approval of the Animal Care and Use Committee, Office of Graduate School and Research, University of Guam. No animals were harmed in this study as interactions were purely *in situ* observations. Fieldwork was carried out under Scientific License numbers 08-001 and 08-003 issued by the Guam Department of Agriculture.

### Study Species and Survey Sites

On the U.S. Pacific island territory of Guam, the thumbprint emperor, *Lethrinus harak*, is heavily targeted by hook-and-line, gillnet, and spear. It remains one of the few numerically abundant, carnivorous fishes encountered on the reef flats. Currently, there are no restrictions on the size or numbers of *L. harak* landed by Guam fisherman. Instead, the primary fisheries management tool is a network of five marine reserves, established in 1997 in response to dramatic declines in catch-per-unit-effort for this and other reef fish species.

This study was conducted at four enclosed reef-flat sites including two marine reserves and two comparable fished sites on Guam (13°50′ N, 144°45′ E). These include Piti Marine Preserve and East Agaña Bay to the west and Achang Marine Preserve and Rios Bay to the south (Figure 1). The Piti and Achang Marine Preserves were established in May 1997 but not fully enforced until January 2001. At certain times of the year, limited shore-based fishing for juveniles of certain reef fish species (rabbitfish, goatfish and trevally) is allowed, with a permit, using cast-nets and hook-and-line.

Study sites ranged in size from 0.9 to 1.7 km^2^, and shared similar biotic and abiotic characteristics. Each site contained comparable fish assemblages and habitat types, and is bordered by fringing reef slopes and high-energy reef crests on the seaward boundary, occasionally bisected by small channels with strong tidal currents. Extensive seagrass beds (*Enhalus acoroides* and *Halodule uninervis*), which are known to be settlement habitat for *L. harak*, occurred throughout all sites. The average depth is less than two meters whereas Piti Marine Preserve also contained a small section of deeper lagoonal habitat (≤10 m) formed by natural limestone sinkholes and dominated by reef-building *Porites* corals.

Digital benthic maps [26] were used to quantify the proportions and total areas of benthic habitat types at each site. The benthic classifications described by Burdick [26] were compressed into six categories: aggregate reef, coral pavement, macroalgae, seagrass, turf pavement, and sand (Figure 2; Table S1).

### Surveys

Underwater visual surveys (by snorkel) were conducted between June and October 2007 using an optimal stratified-random sampling design. For this method, a population that exhibits a patchy distribution in space is divided into a number of subpopulations in which the individual variances surrounding the mean fish densities are minimized. With better stratification, the lower the number of sampling replicates required for confident estimates of mean density [27]. By accounting for area, these estimates can then be pooled to generate a precise estimate of total abundance [28].

The allocation of sampling effort among habitat types was determined using the formula [29],
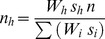
(see Table 1 caption for definition of terms). The model optimizes the total abundance estimate of *L. harak* by allocating sampling replicates to habitats of greater variance in fish density and total area. Standard deviations (s*_h_*) used in the model were obtained from pilot surveys at each site, where five transects were randomly performed in each habitat type (Piti, n = 30; East Agaña, n = 25; Achang, n = 20; Rios, n = 20). The number of transects per site was chosen so that ∼1.5% of the total site area was covered. Individual *L. harak* were counted along 50×5 m transects and allocated to 3 cm size classes. Only one observer (BMT) conducted the fish surveys and trained in underwater size estimation following Bell et al. [30]. All surveys were conducted during morning hours (∼8∶00 am to ∼11∶00 am) and within ±2 hours of the high tide. To avoid transects overlapping, their positions were chosen randomly within a habitat type and marked with a GPS unit.

**Table 2:** Total population size, biomass and spawner biomass estimates of *L. harak* for each site, calculated from stratified visual surveys.

	Marine Preserves		Fished Areas	
	Piti	Achang	East Agaña	Rios
Population estimate (±95% CI)	10720 (2247)	48561 (9346)	18327 (7688)	6876 (4953)
Total biomass (kg) (±95% CI)	2205 (580)	4737 (1667)	477 (318)	535 (434)
Spawner biomass (kg) (±95% CI)	1999 (570)	3578 (1555)	119 (150)	218 (219)

**Table 3:** Results of the nested ANOVA and *post hoc* Tukey comparisons of *Lethrinus harak* population estimates on Guam.

	Density					Adult Density			
Source	*df*	MS	F	p	Post hoc	MS	F	p	Post hoc
Status	1	28.04	10.46	0.001	Prot > Fished	10.34	23.51	<0.001	Prot > Fished
Site(Status)	2	30.51	11.39	<0.001	Ach > Eab, Pi, Ri	2.40	5.46	0.005	Ach >Pi > Ri, Eab
Habitat	3	54.82	20.46	<0.001	SG > SD, MC, TP	0.44	1.00	0.396	NS
Status*Habitat	3	7.78	2.91	0.035		0.47	1.07	0.362	
Error	253	2.68				0.44			
	**Biomass**					**Spawner Biomass**			
**Source**	***df***	**MS**	**F**	**p**	**Post hoc**	**MS**	**F**	**p**	**Post hoc**
Status	1	5573.81	28.76	<0.001	Prot > Fished	4160.55	25.14	<0.001	Prot > Fished
Site(Status)	2	1836.69	9.48	<0.001	Ach >Pi > Ri; Ach > Eab	708.36	4.28	0.015	Ach >Pi > Eab, Ri
Habitat	3	1150.98	5.94	0.001	SG > TP	243.50	1.47	0.223	NS
Status*Habitat	3	284.93	1.47	0.223		107.97	0.65	0.582	
Error	253	193.80				165.49			

Only significant comparisons of *post hoc* tests are presented. NS  =  not significant, Prot  =  Protected, Ach  =  Achang Marine Preserve, Eab  =  East Agaña Bay, Pi  =  Piti Marine Preserve, Ri  =  Rios Bay, SG  =  seagrass, SD  =  sand, MC  =  macroalgae, TP  =  turf pavement.

### Data Analysis

The total population abundance of *L. harak* and associated variance was calculated for each site (Table 1). Site-specific total biomass was calculated using individual fish weights and associated error computed from the length-weight relationship for *L. harak* [21]. Spawner biomass was calculated as the sum of the weight of all reproductively mature individuals:
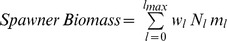
where *N_l_* represents the total number of individuals in size class *l*, *w_l_* represents the mean weight for individuals of size class *l*, and *m_l_* is the proportion of mature individuals in size class *l* (following a logistic curve). Length-to-weight conversions assumed fish were the mean length of their respective 3-cm size class.

Statistical comparison of mean density, adult density, biomass, and spawner biomass were made using a three-factor, two-level nested analysis of variance (ANOVA). Main factors in the ANOVA model included ‘protection status’ and ‘habitat’ and a third factor ‘site’ was nested within protection status (all factors fixed; [31]). For habitats that did not occur at all sites (aggregate reef occurred only at Piti and coral pavement occurred only at Piti and East Agaña), the data were excluded from the analysis. A *post hoc* Tukey test was used to compare means among sites. Data were square-root transformed to conform to assumptions of normality and homoscedasticity.

Mean fork length of *L. harak* was compared among sites using a single-factor ANOVA on natural log-transformed data with a *post hoc* Tukey test. Mean fork length was compared among protection statuses and habitats using a nested ANOVA as before with factors ‘protection status,’ ‘site’ (nested within protection status), and ‘habitat’. Only habitats macroalgae and seagrass were used in the analysis as these were the only habitats where fish were always observed.

**Figure 3:**
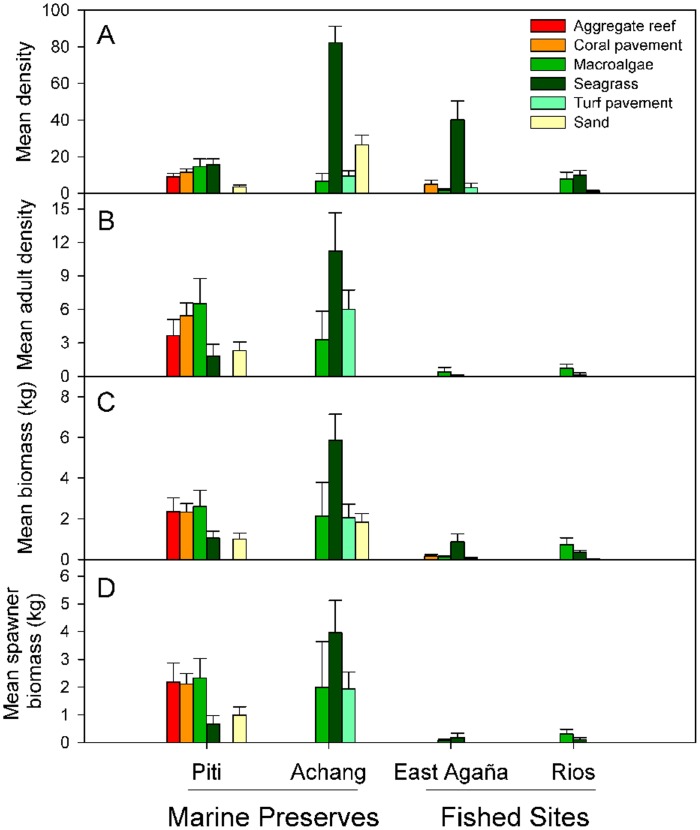
Mean density, adult density, biomass and spawner biomass of *Lethrinus harak* by habitat type at each study site. Values for panels (A) and (B) are per 1000 m^2^ and error bars represent standard error. Note that the absence of bars may not represent zero values as some habitats did not occur in certain sites (see Table 1).

### Model Structure

We used an age-structured population model to estimate changes in population size, spawning potential, age composition, and sex ratio over time within Achang Marine Preserve under scenarios of reserve removal and maintenance. The site was modeled as a single population as previous work shows immigration and emigration of *L. harak* at this scale is unlikely due to small home range size [25] and impermeable natural barriers [32]. The population size and structure in Achang Marine Preserve were assumed near equilibrium. The number of individuals per age class was computed as
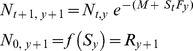
where *N_t,y_* represents the number of individuals of age *t* in year *y*. The parameter *M* is natural mortality (constant across age classes and calculated using age-based catch curves from fish caught at Achang [21]), *F_y_* is the fishing mortality rate in year *y*, and *S_t_* is the fishing selectivity at age *t*. Fishing selectivity was estimated using backwards extrapolation of length-converted catch curves [33], [34] from historical creel survey data (Guam Division of Aquatic and Wildlife Resources, unpub data) and was described by a logistic equation. The number of individuals in age class 0 in year *y +*1 (*N_0,y +_*_1_) is a function of the total spawner biomass in year *y* (*S_y_*), which is equal to the number of recruits in year *y +*1 (*R_y +_*_1_). The age structure of the population for the initial year (year 0) was simulated using the population estimate and the natural mortality rate from Taylor and McIlwain [21].

Total spawner biomass (*S_y_*) for the population for year *y* was modeled as
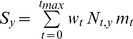
where *t_max_* is the maximum age in the population (13 years; [21]), *w_t_* is the average weight of individuals at age *t*, and *m_t_* is the proportion of individuals mature at age *t*. Maturity at age *t* (*m_t_*) is described by a logistic equation fitted to empirical data [21]. The mean weight-at-age (*w_t_*) was calculated as



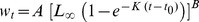
where *L_∞_* (mean asymptotic fork length), *K* (growth coefficient), and *t_0_* (theoretical age at which fork length equals zero) are parameters of the von Bertalanffy growth function, and *A* and *B* are empirically fitted scaling parameters of the length-weight relationship.

**Figure 4:**
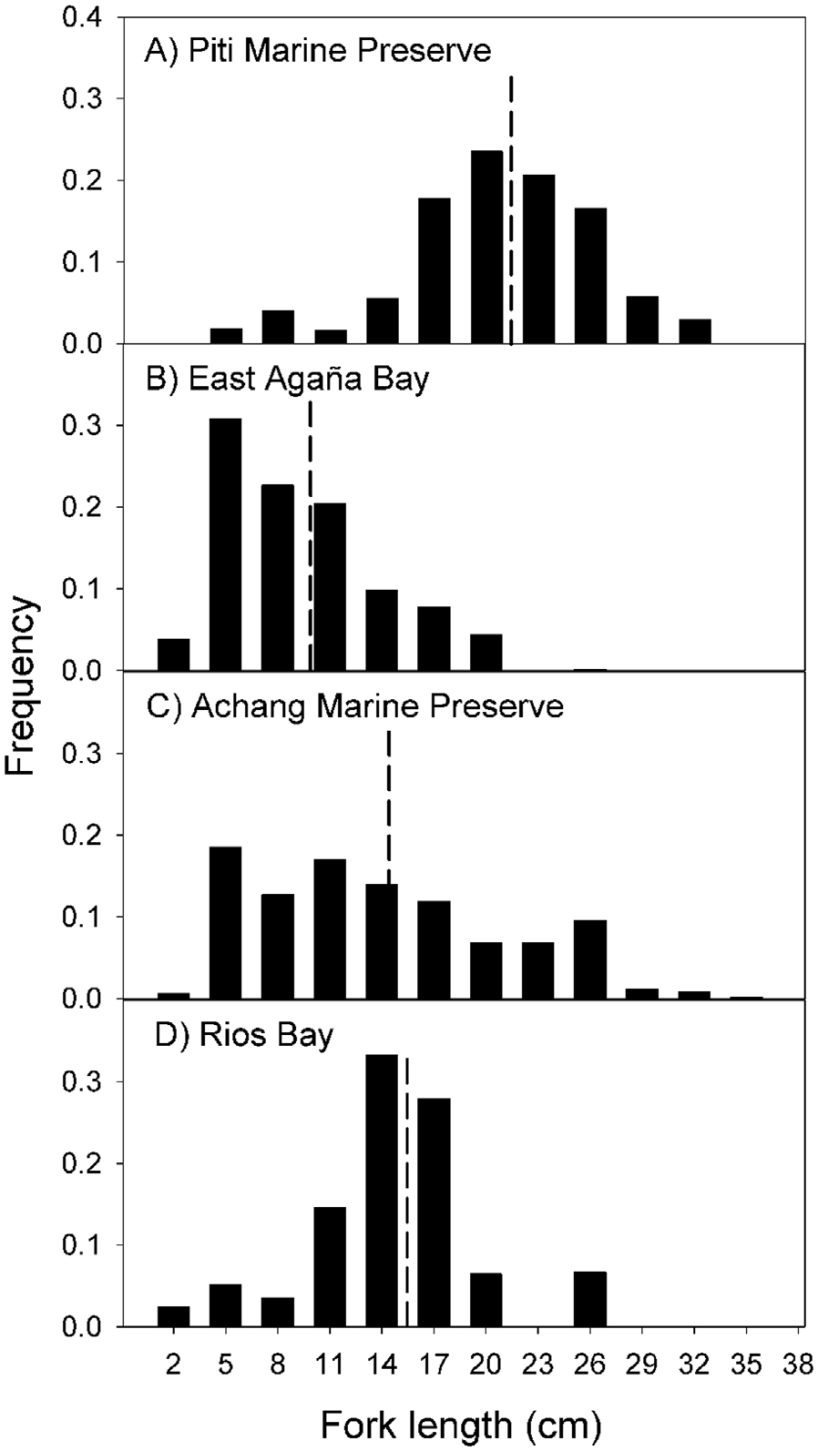
Total size frequency distributions of *Lethrinus harak* for A) Piti Marine Preserve, B) East Agaña Bay, C) Achang Marine Preserve and D) Rios Bay. Data collected from stratified-random visual surveys combined proportionally across habitat types. Dashed lines indicate mean fork length for each site.

**Table 4:** Results of the nested ANOVA and *post hoc* Tukey comparisons showing the influence of protection status, site within protection status and habitat on the mean fork length of *Lethrinus harak*.

Source	*df*	MS	F	p	Post hoc
Status	1	5.09	20.70	<0.001	Prot > Fished
Site(Status)	2	0.40	1.63	0.197	NS
Habitat	1	21.64	88.01	<0.001	MC > SG
Status*Habitat	1	0.16	0.65	0.420	
Error	342	0.25			

Only significant comparisons of *post hoc* tests are presented. NS  =  not significant, Prot  =  Protected, MC  =  macroalgae, SG  =  seagrass.

Recruitment was estimated from total spawner biomass using the Beverton-Holt stock recruitment function [33] where *R_y +_*_1_ represents the number of recruits in year *y +*1.
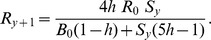



*R_0_* and *B_0_* represent the recruitment and spawner biomass from an unexploited population, respectively, and *h* is the steepness parameter describing the rate at which mean recruitment asymptotes with increasing spawner biomass [35]. Values for the steepness parameter (*h*) were derived based on evaluation of recovery trajectories. Four spawner-recruit relationships were employed and their appropriateness was examined by comparison of resultant population recovery rates with those of empirical data. The four relationships were derived from the Beverton-Holt recruitment model where (1) *h*  = 0.8, (2) *h*  = 0.6, (3) *h*  = 0.4, and (4) a strong depensation effect exists for recruitment at critically low levels of spawner biomass (Figure S1).

**Figure 5:**
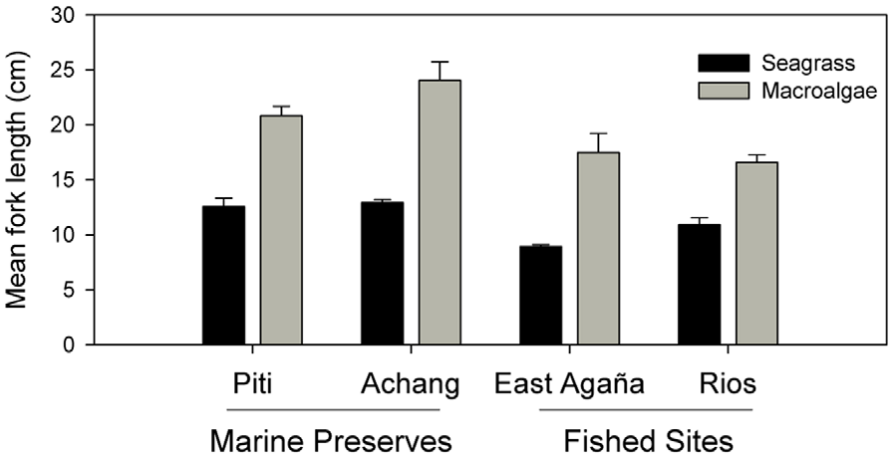
Mean fork length of *Lethrinus harak* by habitat type (seagrass and macroalgae) and protection status at each of the four study sites. Error bars represent standard error.

**Figure 6:**
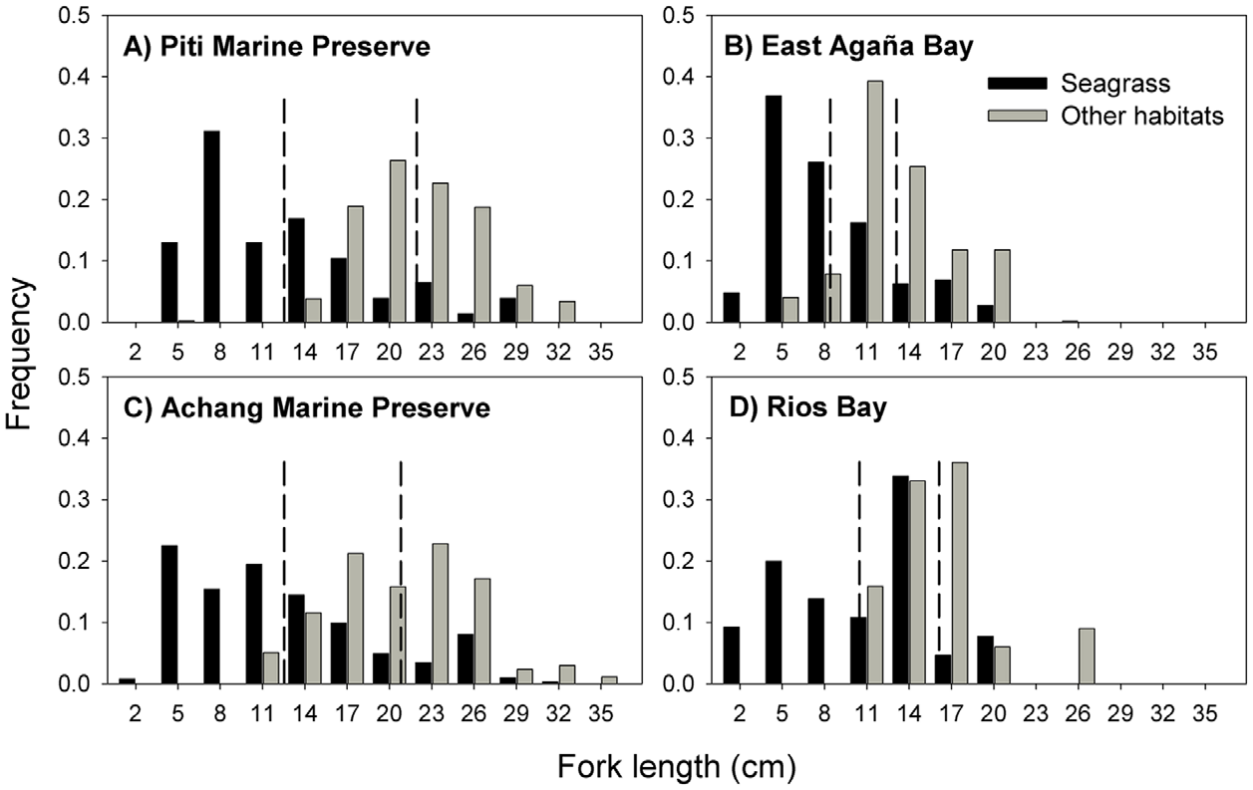
Size frequency distributions of *Lethrinus harak* for seagrass and all other habitats (combined) at each site. Data collected from stratified-random visual surveys. Dashed lines indicate mean fork length.

For each spawner-recruit relationship, total biomass in Achang Marine Preserve was reduced to 10% of the unfished biomass and recovery trajectories were modeled. Population doubling times (*T_d_*) were estimated from the recovery trajectories and the intrinsic rate of population increase (*r*) was calculated using the following formula:
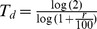



The resultant *r* values were compared with those calculated from recovery trajectories in Russ and Alcala [36] and Russ et al. [37] for Apo Island Reserve (11 and 19 years protection), Sumilon Island Reserve (9 and 9 years protection), and for inferred rates across 13 independent marine reserves in the Philippines (max 13 years protection). Data from Russ and Alcala [36] and Russ et al. [37] are pooled biomasses of all large predatory reef fish which included *L. harak* at Sumilon reserve. These studies represent one of the best datasets on population recovery rates in marine reserves on coral reefs and the areas examined contain similar species assemblages to those in Guam.

Values of *r* from the Philippine marine reserves ranged from 0.13 to 0.28 (Table S2). Hence, in the present study, steepness values of 0.6 and 0.8 yield what are likely the most realistic rates of population increase (*r*  = 0.23 and 0.33, respectively). Stock-recruitment functions with *h*  = 0.4 and a depensation effect yielded considerably low *r* values (*r*  = 0.12 and 0.09, respectively) which are unrealistic given that *L. harak* has a slightly faster turnover rate than most of the species that were pooled together in the Philippines. For this reason, these values were not included in further simulations.

For protogynous species like *L. harak*, size-selective fishing of larger individuals can result in skewed sex ratios with a female bias [38]. We investigated possible changes in the population sex ratio under different fishing scenarios by modeling sex change as a logistic function of age with the proportion of males at age *t* (*X_t_*),
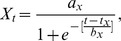
where *a_x_* is the asymptotic sex ratio with age, *t_x_* is the age at *a_x_*/2, and *b_x_* is the slope of sexual transition. Sex change remained fixed regardless of changes in population structure, i.e. there were no compensatory mechanisms controlling the rate of sexual transition [39]. The same logistic function was used to model maturity at age *t* (*m_t_*) and selectivity at age *t* (*S_t_*) with parameters *a_m_*, *t_m_*, and *b_m_* for maturity and *a_s_*, *t_s_*, and *b_s_* for selectivity.

**Table 5:** The description and values for each parameter used in the age-structured population model.

Parameter	Value	Description
*M*	0.243	Natural mortality rate (yr^−1^)*
*F*	0.000, 0.636	Fixed fishing mortality rate (yr^−1^)*
L_∞_	293	Mean asymptotic fork length (mm)*
*K*	0.198	Growth coefficient (yr^−1^)*
*t*_0_	−1.494	Theoretical age when length = 0 (yr)*
*A*	9×10^−6^	Weight-at-age coefficient*
*B*	3.123	Weight-at-age exponent*
*h*	0.600, 0.800	Steepness of spawner-recruit function
*R*_0_	13 090	Recruitment from unexploited population**
*B*_0_	3578	Spawner biomass from unexploited population (kg)**
*a_x_*	0.623	Asymptotic sex ratio of logistic sex transition***
*b_x_*	0.580	Slope of logistic sex transition***
*t_x_*	4.440	Age at half *a_x_* (yr)***
*a_m_*	1	Asymptotic proportion mature*
*b_m_*	0.569	Slope of logistic maturity*
*t_m_*	3.750	Age at 50% maturity (yr)*
*a_s_*	1	Asymptotic selectivity*
*b_s_*	0.644	Slope of logistic selectivity*
*t_s_*	1.88	Age at 50% selectivity (yr)*
*t_max_*	13	Maximum age in population (yr)*

Source of values indicated by asterisk:

* [21];

** present study;

*** [68].

**Figure 7:**
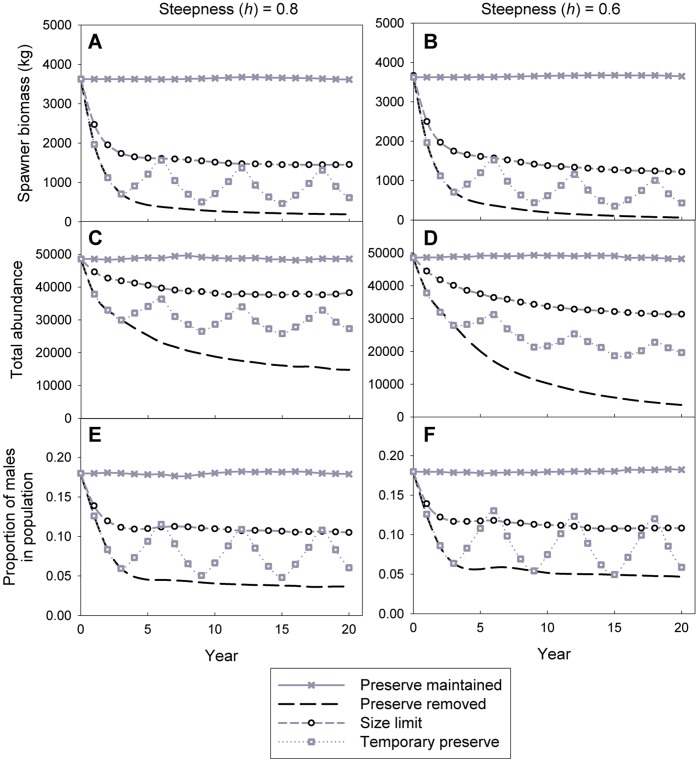
Model projections of various population parameters. The change in spawner biomass, total population abundance, and proportion of males for *Lethrinus harak* within Achang Marine Preserve over twenty years under four management scenarios and two spawner-recruit steepness values. Data are mean outputs of 1000 model simulations for each scenario.

### Model Scenarios

Recent legislation proposed in 2008 and 2009 called for permanently altering Guam’s current marine reserve network which consists of five reserves constituting 11% of the total coastline. These proposals included the complete removal of individual reserves or introducing rotational harvest. Model scenarios for the Achang Marine Preserve include (1) maintenance of current regulation (no extraction of *L. harak*), (2) complete removal of regulations (unrestricted fishing allowed), (3) fishing allowed with minimum size limit (23 cm; [21]), and (4) establishment of rotational reserves (fishing allowed and prohibited at 3 year intervals). A mean fishing mortality of 0.636 yr^-1^ (calculated from neighboring site Rios; [21]) was used to model fishing effort for all scenarios where fishing is allowed.

Rotation of the intervals of opening and closure of protected areas is often proposed as a 1-to-1 time-interval ratio [40], [41], although ratios of the rates of biomass depletion and recovery in coral-reef fish do not conform to such patterns [42]. Hence, the effects of rotational closures was explored by measuring the proportional recovery time of spawner biomass (compared to an assumed pristine state) over all combinations of intervals using the age-structured population model following a given period (up to 20 yrs) of unrestricted fishing.

**Figure 8:**
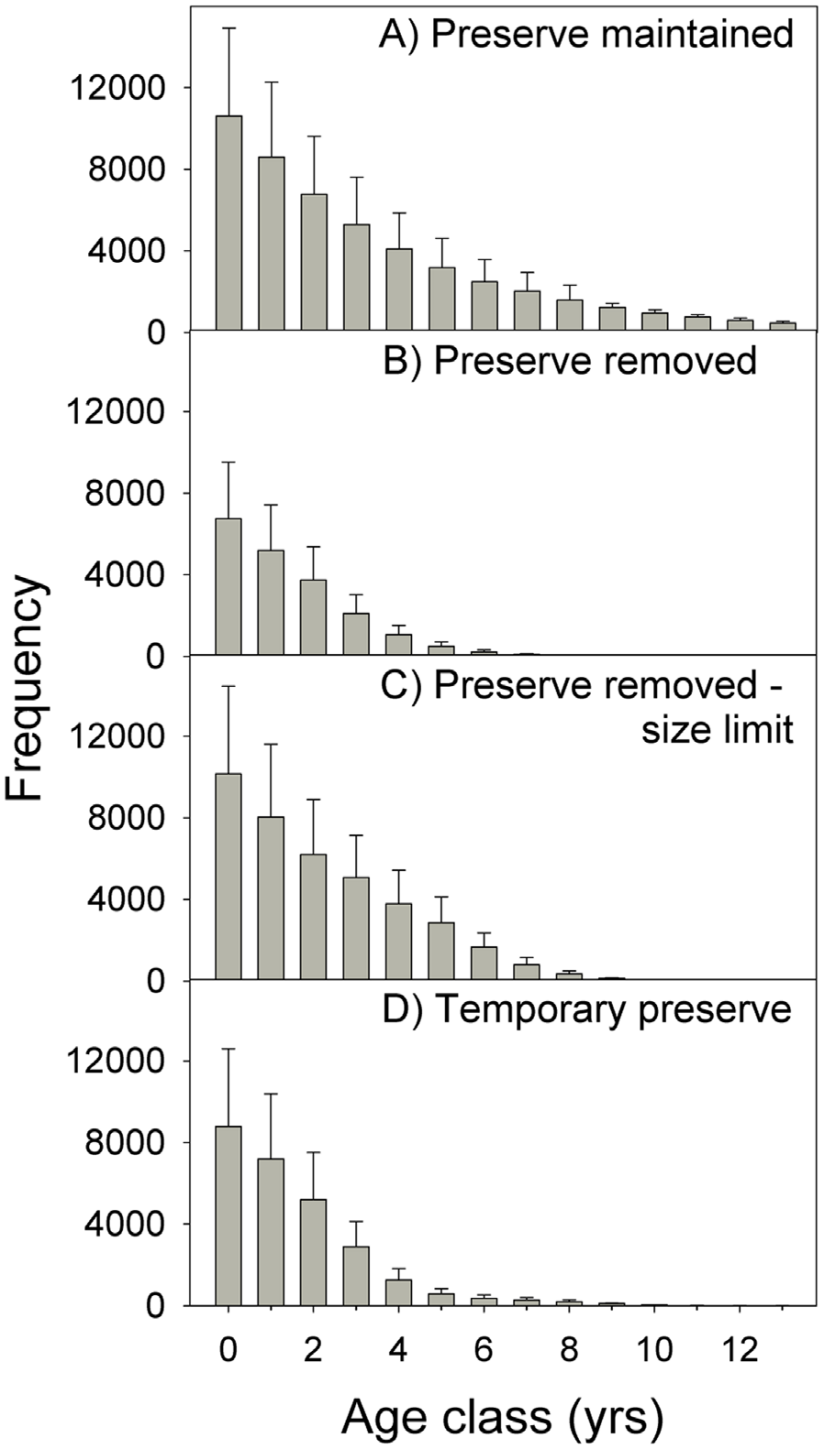
Projected age-frequency distributions. The effect of marine preserve maintenance, removal, and modification on population age structure of *Lethrinus harak* within Achang Marine Preserve after ten years of management. Error bars represent standard deviation of the mean estimate after 1000 random simulations.

**Figure 9:**
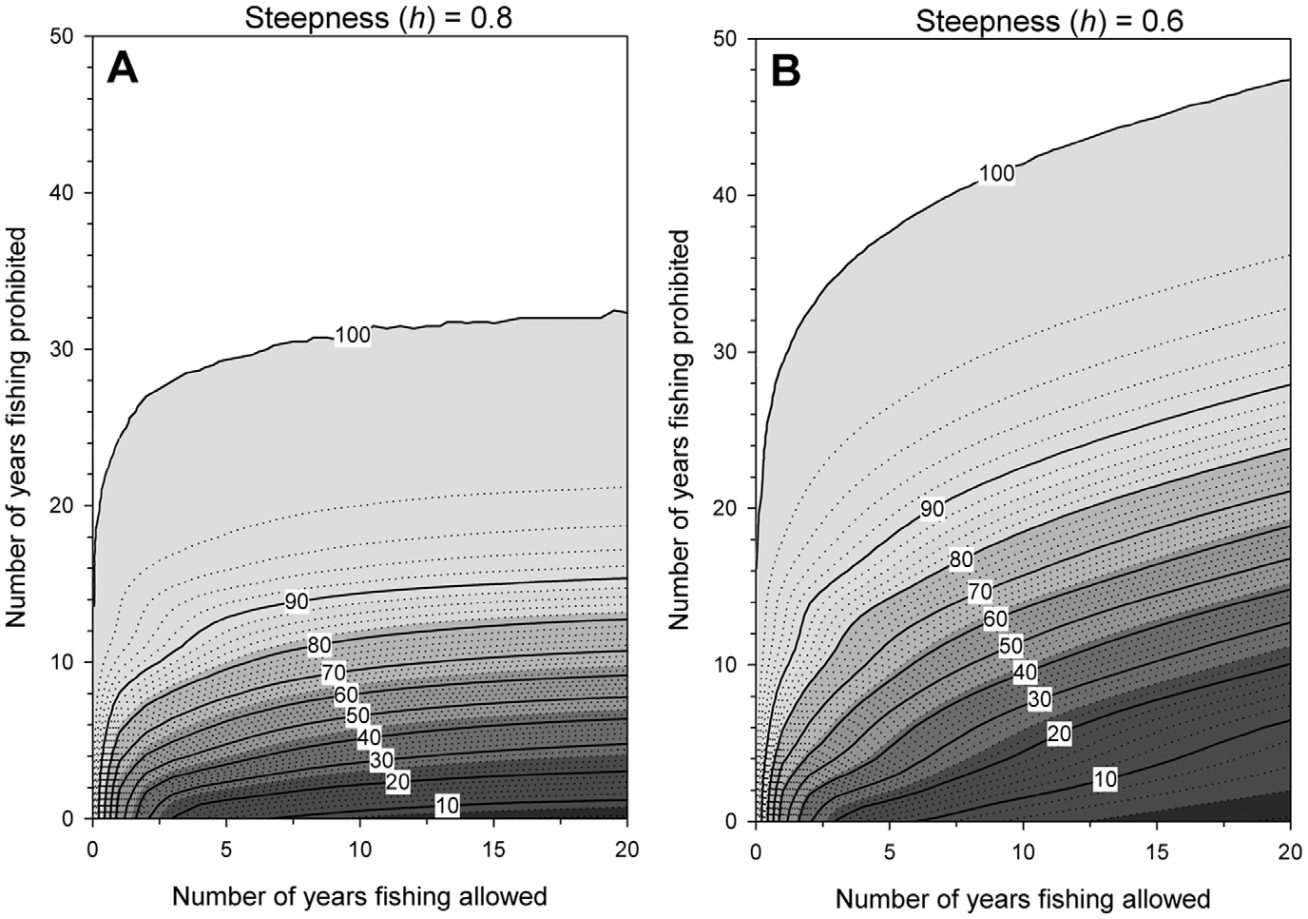
Projected recovery trajectories. The time (years) required for replenishment of spawner biomass (y-axis) in Achang Marine Preserve proportional to an assumed pristine state following a given period of unrestricted fishing (x-axis, up to 20 years). This is presented for two spawner-recruit steepness values, A) *h*  = 0.8 and B) *h*  = 0.6. Contour lines represent the proportional biomass relative to a pristine state (for example: in B, after 10 years of unrestricted fishing, ∼6% of biomass remains compared to a pristine level. After 10 subsequent years of protection, the proportion has risen to 40% of pristine).

## Results

### Abundance, Density and Biomass

The total numbers of *L. harak* counted on 316 transects were 243 (Piti Marine Preserve), 667 (East Agaña Bay), 772 (Achang Marine Preserve), and 100 (Rios Bay). Habitat-specific densities and total abundance estimates are presented in Tables 1 and 2. The population size in Achang ranged from nearly 3 to 7 times greater in comparison to other sites (Table 1). Achang also had the highest overall density (34.6 per 1000 m^2^) followed by the fished sites East Agaña (10.6) and Rios (7.4). Piti had the lowest mean density of 7.0 per 1000 m^2^. The highest habitat-specific mean density (x_h_) of *L. harak* was in seagrass habitat, with as many as 20 individuals/250 m^2^ recorded from Achang (Table 1). While this habitat represented only 20% of the total area across all sites, nearly 70% of the estimated population was recorded there.

Total biomass of *L. harak* from protected sites was, on average, over five times greater than comparable fished sites (Table 2). For spawners, these differences were even more pronounced with 16 times more biomass in protected sites (Table 2). The structure of total biomass varied considerably by protection status. Approximately 91% and 76% of the total biomass, respectively, in the reserves Piti and Achang were comprised of mature fish, whereas in the unprotected sites, Rios and East Agaña, reproductive fish constituted only 41% and 25% of the total biomass, respectively (Table 2).

Mean density, adult density, biomass and spawner biomass were greater within protected sites (Table 3; Figure 3). However, there were strong site-specific differences which must be considered in comparisons at the status level. Achang had significantly greater values than all other sites, whereas Piti exceeded both fished sites only for adult density and spawner biomass (i.e., when juveniles are removed; Table 3). Mean density and biomass differed among habitats because of high numbers of juvenile fish in seagrass (Table 3). The significant interaction between factors ‘status’ and ‘habitat’ for mean density reflects differences in turf pavement driven by Piti (Table 3; Figure 3).

### Size Structure and Mean Size

Overall, Piti had more large fish (>20 cm) than its comparable fished site East Agaña Bay (Figure 4). The mean fork length (21.2 cm) at Piti was twice that of East Agaña (9.7 cm; F_1,908_ = 483.00, P<0.001). Conversely, at the southern sites, there were no differences in the size distributions or mean fork lengths (14.3 and 15.2 cm, respectively) at Achang Marine Preserve and Rios Bay (F_1,870_ = 3.41, P  = 0.065). When we considered habitat, the observed differences in mean fork length among sites were dependent on the proportion of habitat present at that site (Table 4). Fish found in seagrass were mostly small and immature (<15 cm), compared to those from macroalgae which were nearly 5 cm larger (Table 4; Figure 5). At the site level, *L. harak* within seagrass were always smaller when compared to fish from other habitats (Figure 6). East Agaña had the greatest number of juvenile fish with 40% of those in seagrass less than 5 cm in fork length (Figure 6B). A clear pattern of ontogenetic shift in habitat use was evident from plots of size frequencies, with fish below 10 cm rarely encountered outside of seagrass. In contrast, *L. harak* found in non-seagrass habitat were on average seven centimeters larger.

### Population Model

Model parameters and values are presented in Table 5. The response of the *L. harak* population through time varied considerably depending on the management scenario (Figure 7). For scenario one (current marine preserve status maintained), spawner biomass, total abundance, and sex ratio remained stable over time (Figure 7). Scenarios two (marine preserve removed), three (marine preserve removed – minimum size limit enforced), and four (3-year rotational closure established) all yielded sharp declines in spawner biomass immediately after fishing was allowed (Figure 7A-B). For scenario two, nearly 50% of the spawner biomass was removed in the first year, then leveled off at 5% of the unexploited value after 10 years (Figure 7A-B). Similarly, average spawner biomass for scenarios three and four declined to 40% and 30% of the unexploited value, respectively (Figure 7A-B).

The response of total abundance to the four management scenarios was similar to, yet less pronounced than spawner biomass. However, the lower steepness value of the spawner-recruit relationship (*h*  = 0.6) amplified the rate of decline in abundance, particularly when the preserve was removed (Figure 7D). Under this scenario there were less than 5000 fish left 20 years after opening the preserve; a decline of over 90%. The model predictions for changes to the sex ratio were similar to the patterns for spawner biomass and total abundance, with little effect from the steepness values (Figure 7E-F).

We investigated how the age structure of *L. harak* changed by plotting the mean age-frequency distribution ten years after the management scenarios were introduced (Figure 8). Mean ages for the population under each scenario are 3.2 years (scenario one), 1.4 years (scenario two), 2.2 years (scenario three), and 1.6 years (scenario four) (Figure 8). After ten years, individuals greater than 8 years old represented 33% of the reproductive population by number and 48% by biomass when the marine preserve is maintained (scenario one). For the three alternative scenarios, the older age classes were severely truncated or removed entirely. Under scenario two, older individuals represented 4% of reproductive abundance and 8% of reproductive biomass. For scenarios three and four, these values were 6% (abundance), 11% (biomass), 13% (abundance), and 24% (biomass), respectively.

The recovery time for spawner biomass when fishing is unrestricted is much slower than the rate of depletion (Figure 9). Further, while steepness (*h*) of the spawner-recruit function had little impact on the patterns of population parameters under the four management scenarios (Figure 7), it had a significant effect on recovery trajectories (Figure 9A-B). When recruitment is more dependent on the spawning stock in Achang (*h*  = 0.6), recovery times increase considerably, especially after longer periods of fishing are permitted (Figure 9B).

## Discussion

Since the early 1980s, when detailed fishery data was first collected on Guam, *L. harak* has been among the most heavily fished species [43]. In that time, there has been increasing evidence of persistent overfishing on Guam’s coral-reef fishery [44], [45]. Hence, the demographic differences between sites open and closed to fishing in this study are not surprising. While the distribution of benthic habitat varied among sites, these differences did not explain the considerable discrepancies observed for spawner biomass and adult density. Instead, our data suggest these were attributed to protection from fishing rather than underlying habitat effects. Considering the magnitude of the differences in both total biomass and spawner biomass between marine reserves and comparable fished sites, it is clear Guam’s marine reserves make a disproportionate contribution to the island-wide reproductive potential of *L. harak*.

The maintenance of a reproductively viable stock is the paramount conservation and fishery management objective [46]. While differences in mean density and biomass across protection statuses were somewhat indistinct, adult density and spawner biomass were an order of magnitude greater in protected sites. Spawner biomass at Achang Marine Preserve greatly exceeded all other sites. Piti also demonstrated a high reproductive potential, despite having a relatively low total abundance. Conversely, spawner biomass and adult density estimates for East Agaña were based on only two individuals above the size at maturity recorded from ninety independent transects. While increased biomass of target species within protected sites is frequently demonstrated in the primary literature [47], [48], [49], [50], the magnitude of difference between protected and fished sites in our study is among the highest estimates published to date (reviewed in [5]). This is likely a result of Guam’s history of intense exploitation of marine resources and the heavy reliance on unsustainable fishing practices such as monofilament gill netting [43]. In addition, ratios of spawner to total biomass differed considerably between levels of protection status, giving further indication that the size-selective effects of fishing have had a major impact on the demographic signature of these populations. The benefits of increased reproductive potential within protected sites to adjacent areas are logistically difficult to demonstrate *in situ* [5], and empirical evidence of enhanced larval transport for coral-reef fishes is sparse. However, these proposed benefits have been established via modeling [1], [11], [51], [52] which show that, given appropriate environmental and biological conditions, exploited fish populations can be significantly enhanced through larval export.

A decrease in mean fish size has been considered one of the primary indicators of overfishing or stock decline [53], [54]. Numerous examples exist where mean fish size increased in protected areas after their establishment [55], [56]. Our results, however, serve as an example of why and how interpretation of mean sizes should be done carefully. For example, such statistics can be easily confounded by habitat variability and the inherent distribution of size structure within a population caused by ontogenetic shifts in habitat preference. Here, overall mean size in Achang Marine Preserve was no different than that of Rios Bay. However, at the habitat level, mean size in Achang was consistently greater; indicating that comparison of overall mean size was confounded by underlying habitat distributions. Such differences would not have been detected with simple random sampling and were only detected because of the stratified nature of our experimental design. Additionally, mean size estimates can be influenced by recruitment variability which could over- or under-represent the smaller size classes [57]. It is difficult to make conclusions regarding recruitment variability between sites without a recent time-series of recruitment data. But irrespective of any effects of recruitment strength on size structures, abundance estimates show that there is a severe lack of larger fish within unprotected sites. Therefore, we argue it is unlikely that the observed differences in population structure were driven by recruitment variability.

Results from the population model show that other management scenarios do not produce reproductive benefits compared with those that are evident at fully protected sites. With unrestricted fishing (scenario two), the model produced results similar to empirical data collected at the unprotected sites, which lends support to the accuracy of the model. Further, the effects of alternative management scenarios showed consistent patterns across measures of abundance, spawner biomass, age structure and sex ratio, all of which declined considerably compared with a fully protected state. While stock-recruitment dynamics around Guam are poorly understood, we feel the use of a stock-recruitment function for Achang was justified. The production differential between protected and fished sites and the comparison of modeled recovery rates with those of empirical studies suggests the assumption of self-recruitment is plausible. For example, based on long-term recovery data, Russ and Alcala [36] proposed that significant self-recruitment back to Philippine reserves did not occur until several years after reserve establishment when adult biomass had increased above a certain threshold. In addition, certain assumptions in our model were purposely chosen to be conservative, such as the fixed fishing mortality rate and the equilibrium carrying capacity. The effect of opening a marine reserve on Guam to fishermen would likely draw the interest of fishermen at a magnitude that would yield a much greater fishing mortality (especially in initial years) than has been calculated at adjacent sites. Also, the model assumes that the Achang population is near carrying capacity but this value is likely higher given that surveys were conducted after seven years of full protection, which is roughly half the maximum lifespan of *L. harak* [21], [22]. Hence, it is plausible the model predictions we derived would in fact be more pronounced [58]. Furthermore, while changes in sex ratio were modeled, we did not explore their potential flow-on effects in any detail. Brooks et al. [59] tested the effects of decreased fertilization rates in protogynous populations where males are more heavily targeted and demonstrated that size-selective fishing effects have impacts on these populations that go beyond simple declines in spawner biomass.

Single-species management has unjustly received considerable criticism (see [60], [61]) and opting for an ecosystem modeling approach can overlook important details of a species’ population biology. In fact, multispecies approaches, though insightful, rarely recognize differences in a species’ response to various management scenarios. For example, Kleczkowski et al. [62] found that reef-fish community assemblages did not differ between protected and fished sites, despite significant patterns identified for certain targeted species. Here, detailed analysis of *L. harak* serves as a proxy for species with similar life histories in a heavily exploited multispecies fishery. Results from our model are congruent with both empirical evidence [41] and previous models [63], [64]. For instance, by modeling short-term impacts of marine reserves compared with minimum size limits, Bohnsack [63] determined that concerns regarding displacement of fishing effort by reserves were largely unsubstantiated, especially for small reserves like those on Guam. His study did not quantify effects on reproductive potential but did point out that a major difference between management scenarios was that protected areas afford protection to all age classes, thereby allowing natural age structures to accrue within protected boundaries for less mobile species. A similar comparison of short- and long-term benefits of marine reserves, minimum size limits, and rotational closures found that reserves provided greater long-term catches and yielded much larger reproductive potential than minimum size limits [64]. Such enhancement of reproductive potential is particularly important for species that are self-recruiting as it protects against recruitment overfishing. Rotational closures, however, provided no fishery benefit whatsoever, as they had the highest cumulative loss over the short-term and provided no long-term catch enhancement.

Our model also suggests that rotational closures offer no long-term benefit to fish populations and fisheries. Instead, we found rotational closures produced a steady pattern of population decline over time. In fact, we postulate that rotational closures in a heavily exploited fishery completely undermine the natural processes related to reef-fish life histories that allow protected-area management to be effective. Empirical evidence from Hawaii indicates short-term closures do not allow sufficient biomass recovery [41]. Opening and closing the Waikiki-Diamond Head Fishery Management Area (FMA) for more than two decades resulted in a steady population decline similar to our model predictions. In the end, fish biomass in the FMA was no different from that of nearby fully unprotected sites. The use of rotational closures has been supported for sedentary invertebrate stocks [3], [40], [65], but there is no evidence to date of their benefit to reef-fish stocks. Ultimately, historical data and life-history theory suggests that, on coral reefs and elsewhere, what can be harvested in a short time period requires a considerably longer recovery time, especially for longer-lived species. While significant increases in fish biomass are commonly seen within four years of reserve establishment, full recovery times for reef-fish populations have been estimated between 15 and 40 years ([42], [66], [67]; present study).

Finally, because *L. harak* displays life-history characteristics typical of many reef fish and occupies several different habitats throughout its life, we are confident that other comparable species benefit from this reserve network in a similar manner. Given the diversity of life histories, movement patterns, reproductive strategies, and trophic guilds of targeted fishes on coral reefs, no reserve or reserve network is a panacea for all species of concern. On Guam, additional management regulations such as minimum size limits or gear restrictions would supplement reserve protection and likely increase island-wide reproductive potential. This would require additional biological and fishery information for various species as well as ongoing monitoring to evaluate the efficacy of new regulations. Concurrently, future research should focus on species-specific patterns of population connectivity among various sites around the island. Such an approach could combine species’ life histories and spatial modeling based on ocean current patterns and site-specific demography, enabling better understanding of source-sink dynamics and the effectiveness of current reserve placement.

Unfortunately, it is difficult to predict the future impacts of marine reserve networks on adjacent fisheries. Despite the much greater reproductive potential within Guam’s reserves, there are still uncertainties regarding stock-recruitment relationships and connectivity between sites. In addition, we have no pre-protection data on *L. harak* populations at each study site, which limits our conclusions as to the long-term benefits of protected-area management. However, the greater reproductive potential within protected sites highlights their importance as they contribute disproportionately to the reproductive potential of the *L. harak* stock around Guam. This study underscores the utility of exploring single-species population biology and the application of empirical data to models to explore the effects of marine reserves on reef-fish populations.

## Supporting Information

Figure S1 **Spawner-recruit models.** Four potential spawner-recruit models characterizing the relationship between spawner biomass of *Lethrinus harak* in Achang Marine Preserve and subsequent recruitment to the site. When *h*  = 0.8, there is virtually no relationship between spawning stock and subsequent recruitment except at very low levels of spawner biomass. At *h*  = 0.6, this relationship is stronger, indicating that recruitment to Achang Marine Preserve is moderately dependent on the spawner biomass within the site. At *h*  = 0.4, the relationship is very strong. Depensation suggests that there is some critically low level of spawner biomass below which reproductive success is severely hindered and recovery will take much longer than otherwise expected.
(TIF)

Table S1 **Benthic habitat classifications used for stratification of sampling effort.** Six general categories were adapted from Burdick [26].
(DOCX)

Table S2 **Estimates of the intrinsic rate of population increase (** ***r*** **) for model results from the present study and from long-term empirical data from Russ and Alcala** [**36**] **and Russ et al.** [**37**] **.**
(DOCX)
