# Supplementary figures and images for: Marine Reserves and Reproductive Biomass: A Case Study of a Heavily Targeted Reef Fish
Source: PLoS One. 2012 Jun 26;7(6):e39599. doi: 10.1371/journal.pone.0039599 (PMC3383677; doi:10.1371/journal.pone.0039599)

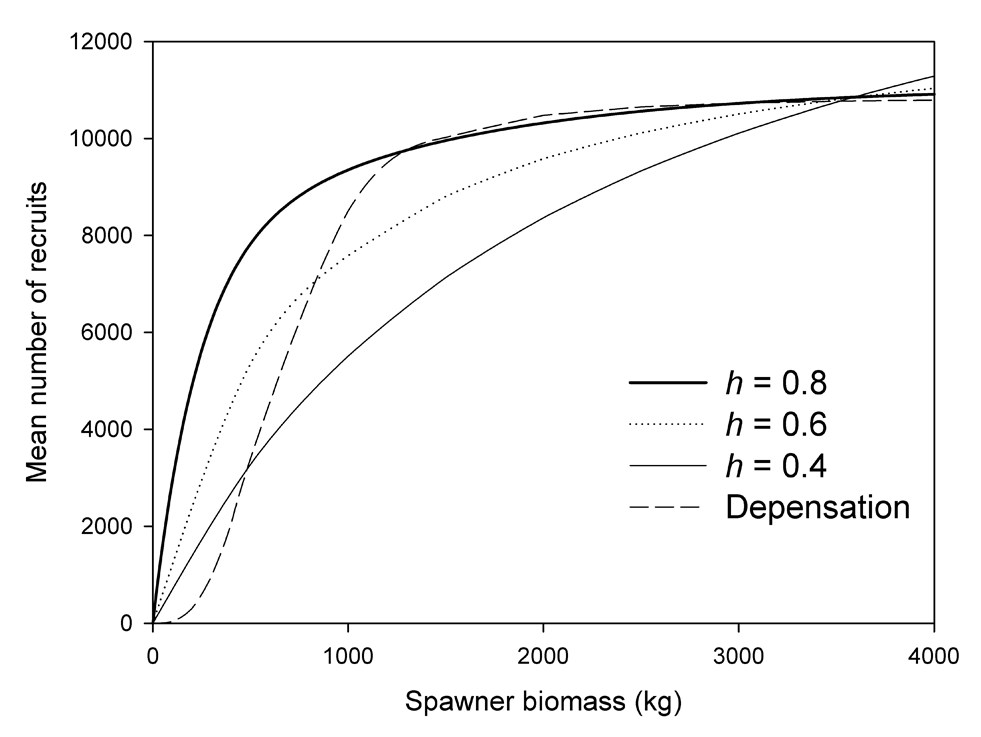

Supplement: Figure S1 — Spawner-recruit models. Four potential spawner-recruit models characterizing the relationship between spawner biomass of Lethrinus harak in Achang Marine Preserve and subsequent recruitment to the site. When h = 0.8, there is virtually no relationship between spawning stock and subsequent recruitment except at very low levels of spawner biomass. At h = 0.6, this relationship is stronger, indicating that recruitment to Achang Marine Preserve is moderately dependent on the spawner biomass within the site. At h = 0.4, the relationship is very strong. Depensation suggests that there is some critically low level of spawner biomass below which reproductive success is severely hindered and recovery will take much longer than otherwise expected. (TIF) [file pone.0039599.s001.tif]
