# Supplementary material for: Marine Reserves and Reproductive Biomass: A Case Study of a Heavily Targeted Reef Fish
Source: PLoS One. 2012 Jun 26;7(6):e39599. doi: 10.1371/journal.pone.0039599 (PMC3383677; doi:10.1371/journal.pone.0039599)
Supplement: Table S1 — Benthic habitat classifications used for stratification of sampling effort. Six general categories were adapted from Burdick [26]. (DOCX) [file pone.0039599.s002.docx]

| **Habitat** | **Description** |
| --- | --- |
| *Aggregate reef* | Aggregate Reef, Coral 10-90%: high relief substrate consisting mainly of reef-building corals dominated by *Porites rus* and *P. cylindrical* at depths from 1-10 m. Found only at Piti Marine Preserve. |
| *Coral pavement* | Pavement, Coral 10-90%: flat, low relief substrate consisting primarily of massive *Porites* spp. as well as other robust reef-flat corals such as *Acropora pulchra*, *Pavona decussata*, and *Pocillopora damicornis* and occurs in depths of 1-3 m. Found at Piti Marine Preserve and East Agaña Bay. |
| *Macroalgae* | Pavement, Macroalgae 10-90%: consists of brown, thallate macroalgae such as *Padina boryana*, *Sargassum polycystum*, *S. cristaefolium* and *Dictyota* spp. in depths ≤ 2 m. Occurs at all sites. |
| *Seagrass* | Seagrass 10-100%: consists of stands of *Enhalus acoroides* (In Piti Marine Preserve, Achang Marine Preserve and Rios Bay) and *Halodule uninervis* (in East Agaña Bay) in depths ≤ 2 m. |
| *Turf pavement* | Pavement, Turf 50-90%: consists of flat, low relief rock or pavement substratum covered by algal turf communities and occurs in ≤ 2 m. Occurs at all sites. |
| *Sand* | Sand, Uncolonized 90-100%: consists of sandy habitats colonized by less than 10% biological cover. Occurs at all sites. |
