# Supplementary material for: Marine Reserves and Reproductive Biomass: A Case Study of a Heavily Targeted Reef Fish
Source: PLoS One. 2012 Jun 26;7(6):e39599. doi: 10.1371/journal.pone.0039599 (PMC3383677; doi:10.1371/journal.pone.0039599)
Supplement: Table S2 — Estimates of the intrinsic rate of population increase ( r ) for model results from the present study and from long-term empirical data from Russ and Alcala [36] and Russ et al. [37] . (DOCX) [file pone.0039599.s003.docx]

| **Data** | **Estimate of** $\boldsymbol{r}$ |
| --- | --- |
| Present study: *h* = 0.8 | 0.33 |
| Present study: *h* = 0.6 | 0.23 |
| Present study: *h* = 0.4 | 0.12 |
| Present study: Depensation | 0.09 |
| Apo Reserve [36] | 0.17 |
| Apo Reserve [37] | 0.13 |
| Sumilon Reserve [36] | 0.20 |
| Sumilon Reserve [37] | 0.23 |
| Inferred across 13 reserves [37] | 0.28 |
